# Reliability modelling and evaluating of wind turbine considering imperfect repair

**DOI:** 10.1038/s41598-023-32575-8

**Published:** 2023-04-01

**Authors:** Panpan Fan, Yiping Yuan, Jianxiong Gao, Yuchao Zhang

**Affiliations:** 1 School of Mechanical Engineering, Xinjiang University, Urumqi, 830002 China; 2 CSSC Haiwei (Xinjiang) New Energy Co., Ltd., Urumqi, 830006 China

**Keywords:** Mechanical engineering, Energy infrastructure, Renewable energy

## Abstract

To model and evaluate the reliability of wind turbine (WT) under imperfect repair, an improved Log-linear Proportional Intensity Model (LPIM)-based method was proposed. Initially, using the three-parameter bounded intensity process (3-BIP) as the benchmark failure intensity function of LPIM, an imperfect repair effect-aware WT reliability description model was developed. Among them, the 3-BIP was used to describe the evolution process of the failure intensity in the stable operation stage with running time, while the LPIM reflected the repair effect. Second, the estimation problem for model parameters was transformed into a minimum solution problem for a nonlinear objective function, which was then solved using the Particle Swarm Optimization algorithm. The confidence interval of model parameters was finally estimated using the inverse Fisher information matrix method. Key reliability indices interval estimation based on the Delta method and point estimation was derived. The proposed method was applied to a wind farm’s WT failure truncation time. The proposed method has a higher goodness of fit based on verification and comparison. As a result, it can bring the evaluated reliability closer to engineering practice.

## Introduction

Wind, as a sustainable and affordable energy source, represents a strong alternative to traditional energy sources. The core concerns for wind turbine (WT) manufacturers and operators are to increase its reliability and decrease costs, therefore enhancing commercial competitiveness^1^. Former US Secretary of Energy and Nobel Prize-winning physicist Steven Chu stated that the operation safety and reliable of WT is an issue that must be resolved continuously^2^. Accurately evaluating the WT reliability during service phase is crucial to ensure their reliable operation and safe service.

WT is a complex electromechanical and hydraulic equipment that can be repaired, and repair will affect its reliability^3^. In most cases, the reliability of repairable equipment is modelled and analysed by fitting the failure time data with a random point process^4^. Different random point processes represent different repair effects. The majority of current studies used Weibull distribution and its modified distribution^5–7^ to model the reliability of WT. The Weibull distribution belongs to the general renewal process, which is believed to restore equipment to as same as new condition, i.e., perfect repair. In fact, WT can be returned to normal operation by performing simple maintenance operations such as adjusting the preload, replacing the lubricating oil, fastening the communication joint wiring, filling the hydraulic oil, and adding coolant. Its repair effect can be regarded as perfect repair. However, replacement and repair of components are more common repair method for WT throughout its service life.

The WT is composed of several components, and any component failure will cause the failure of holistic WT. When one or some components are replaced or repaired, the components are restored as good as new condition, but for the WT as a whole, except for the replaced components, other components still work at varying ages. In other words, the replacement or repair of components will not restore the holistic WT as new. Therefore, Accordingly, from the perspective of the holistic WT, the repair effect is imperfect repair^5,8,9^. The effect of imperfect repair is between that of a perfect repair and a minimal repair. In other words, a repaired equipment exists in a state between "as new" and "as old," which more accurately reflects the reality of WT repair engineering^8^. With the operation and maintenance of WT, the failure rate will gradually decrease during the initial stage of stable operation. With an increase in WT repair times and the accumulation of service time, the normal working time after repair becomes shorter and the probability of failure is increasing^9^. Frequent repair transforms the equipment failure rate into a bounded increasing function so that it approaches a constant value. This is consistent with the notion emphasised by the failure law of complex equipment (e.g., Drenik's law)^3^. There are numerous research results on the reliability modelling and evaluation of repairable equipment under imperfect maintenance effect. Currently, the generalized renewal process (GRP)^10^ and proportional intensity model (PIM)^11^ are the predominant reliability modelling methods based on failure time data.

Kijima et al.^10^ proposed two GRP models, i.e., GRP-I and GRP-II. The model is founded on the "renewal process" and "non-homogeneous Poisson process", and the service age regression factor is introduced to characterise the repair effect in terms of the degree of service age reduction. However, model parameters solutions are complex. Monte Carlo (MC) simulation^12^, maximum likelihood estimation (MLE)^13^, Bayesian method^13^, likelihood ratio test^14^, expectation-maximum algorithm (EM)^15^, nonlinear constrained programming^16^, etc. were proposed as a solution for this issue. Due to the discontinuity of the model, it is challenging for the aforementioned methods to produce a closed-form solution for the reliability indices.

Cox et al.^11^ proposed the PIM, which considered the impact of repair activities on the service reliability of equipment and emphasised the evaluation of equipment repair level by intensity reduction coefficient. To describe the imperfect repair of WT, Zhao et al.^9^ introduced the Weibull proportional intensity model. The cumulative number of system failures was used to represent the repair history. The cumulative failure intensity function was introduced as a covariate in the proportional intensity model (PIM), and the Log-linear proportional intensity model (LPIM) was proposed^17^. Zhang et al.^18^ proposed LPIM to evaluate the reliability of CNC machine tools under imperfect repair conditions. However, LPIM is incapable of describing the bathtub failure process with infant mortality and wear-out failures. In light of this, Zhu et al.^19^ developed the superposed log-linear proportional intensity model (S-LPIM) to model the reliability of the CNC machine tools under imperfect repair conditions, and the model fitted the characteristics of the CNC machine tools failure rate well. In the above PIM model and its improved version, the power law process (PLP) and log-linear process (LLP) are primarily used as benchmark functions of system failure intensity. The common disadvantage of these two process models is that failure intensity increases indefinitely as service time accumulates. In other words, the model lacks a closed solution, which is inconsistent with the engineering practice. After the repairable equipment enters its period of wear-out failure, the repair frequency rises proportionally to the deteriorating reliability. Frequent repair transforms the failure intensity into a function with a finite increase rather than an infinite increase^3^. Accordingly, Pulcini et al.^20^ concluded that repeated repair measures will result in a finite bound of increasing failure intensity for repairable equipment, and the failure intensity will approach a constant as equipment operation time increases. Accordingly, a bounded intensity process (BIP) suitable for estimating the reliability of repairable equipment during its wear-out period was proposed. However, the failure intensity of this model is zero at the beginning of data collection, which is inconsistent with engineering practice. The actual wear-out period is preceded by a period of random failures, during which the failure intensity is not zero. Based on the BIP model, position parameters were introduced to develop the three-parameter bounded intensity process (3-BIP) model^21^, which enabled the accurate evaluation of CNC machine tools reliability. Ren et al.^22^ analysed the impact of running time and repair behavior on equipment reliability and proposed a continuous proportional intensity model with a closed-form solution for modelling and evaluating reliability under imperfect repair. Nonetheless, the equipment failure intensity is zero at the time of early observation period.

The least square method^19^, the MLE^23^, and the Bayesian method^24^ are frequently used to solve reliability model parameters. The first two are statistical methods based on the principle of minimum empirical risk, but when the number of samples is small, the solving accuracy is poor. The Bayesian method is advantageous when the sample size is small, but the estimation accuracy is dependent on the prior distribution chosen. MLE establishes a set of nonlinear transcendental equations by directly deriving the partial derivative of the log-likelihood function and then solves these equations using an analytical method, numerical method, parameter optimization, and other methods. Among them, the analytical method for solving the nonlinear transcendental equations of the multiparameter complex distribution model is the most challenging. For solution, numerical iterative methods such as the Newton method and the EM algorithm are utilised. Improper selection of the primary value will result in iterative process divergence^25^, and it is simple to fall into local optimization. The disadvantages of the preceding MLE algorithm restrict its application to parameter estimation of multi-parameter reliability models. Estimating the parameters of a multi-parameter reliability model is essentially an optimization problem involving nonlinear model parameters. It is feasible to use intelligent optimization algorithms such as particle swarm optimization (PSO) and genetic algorithm (GA) to solve the optimization problems of nonlinear complex functions. The PSO algorithm has few adjustment parameters, a rapid convergence rate, a simple concept, and is straightforward to implement, avoiding the complex operation of GA. PSO algorithm does not depend on the analytical properties of the objective function. It establishes a nonlinear optimization model with constraints, and solves the reliability model parameters by searching the global extreme value and the local extreme value in parallel, which reduce the difficulty of solving the problem.

To sum up, this study has aimed at developing a method for WT reliability modelling and evaluating based on failure time data considering imperfect repair effect. The main contributions of this paper were summarized as follows. (1) The 3-BIP was utilised as the benchmark failure intensity function of the LPIM when constructing the WT reliability model while accounting for the effect of imperfect repair. The 3-BIP was used to describe the evolution of the failure intensity concerning the operation time. The repair effect was described using the LPIM model. (2) The model parameter estimation problem was transformed into a maximum solution problem for a nonlinear objective function. Using the PSO algorithm's capability for global optimization, model parameters are solved by minimising the mean square error. (3) The inverse Fisher information matrix method was used to estimate the confidence interval of model parameters, and interval estimation of the key reliability indices based on the Delta method was derived. (4) The proposed method was validated using actual wind farm WT failure truncation time data. The proposed model goodness-of-fit was evaluated by using the Akaike Information Criterion, Bayesian Information Criterion, and correlative coefficient *R*. The result indicates that the proposed reliability model has higher fitting accuracy. The obtained reliability indices are more accorded with the engineering practice well.

## Methodology

### Reliability modelling for WT with failure time data

The failure intensity function of LPIM is:

1 $$\lambda \left( t \right) = \lambda_{0} \left( t \right)\exp \left[ {\gamma m\left( t \right)} \right]$$

where $\lambda \left( t \right)$ represents the number of failures per unit of time;$\lambda_{0} \left( t \right)$ is the benchmark failure intensity function; $\gamma$ is the repair efficiency factor and $m\left( t \right)$ is the average cumulative number of failures. LPIM believes that each repair activity will affect the equipment’s reliability, thereby altering the failure intensity. $\gamma m\left( t \right)$ represents the cumulative effect of repair. When $\gamma<0$ the repair enhances equipment reliability. When $\gamma = 0$, the repair has no significant effect on the reliability. When $\gamma>0$, the repair degrades the reliability.

The average cumulative number of failures can be expressed as:

2 $$m\left( t \right) = \int_{0}^{t} {\lambda \left( t \right)dt}$$

According to Eq. (1), the closed form of $m\left( t \right)$ and $\lambda \left( t \right)$ can be derived. Rewrite Eq. (2) as:

3 $$\lambda \left( t \right) = \frac{dm\left( t \right)}{{dt}} = \lambda_{0} \left( t \right)\exp \left[ {\gamma m\left( t \right)} \right]$$

With the operation and repair of the WT, the failure rate will gradually decrease during the stable operation phase. After WT enter the wear-out failure period, the frequency of repairs increases as the system's reliability decreases. However, frequent repair transforms the system's failure intensity into a function with a finite increase rather than an infinite one. Consequently, the 3-BIP model^4^ was utilised as the benchmark failure intensity function of LPIM to describe the evolution process of the failure intensity with the operation time. $\lambda_{0} \left( t \right)$ can be defined as:

4 $$\lambda_{0} \left( t \right) = \alpha \left[ {1 - \exp \left( { - \frac{t + \varepsilon }{\beta }} \right)} \right],\quad \alpha ,\beta ,\varepsilon > 0,\,\,\,\,\,t \ge 0$$

where $\alpha$ is the upper limit of failure intensity; $\beta$ is the time that takes for failure intensity to increase from its initial level to the upper limit $\alpha$; $\varepsilon$ represents the change in position of the failure intensity function along the time axis *t*, indicating that the system has been operating for at least $\varepsilon$ at the time of the initial data collection. When *t* = 0,  $\left. {\lambda_{0} \left( t \right)} \right|_{t = 0} = \alpha \left| {1 - \exp \left( { - \frac{\varepsilon }{\beta }} \right)} \right|$ the value represents the actual intensity of failure at the initial moment. When $t \to \infty$, $\left. {\lambda_{0} \left( t \right)} \right|_{t = \infty } = \alpha$.

By substituting $\lambda_{0} \left( t \right)$ into Eq. (1), we can derive:

5 $$\lambda \left( t \right) = \alpha \left[ {1 - \exp \left( { - \frac{t + \varepsilon }{\beta }} \right)} \right]\exp \left[ {\gamma m\left( t \right)} \right]$$

By solving the differential equation, the closed-form solution can be obtained. The differential equation was derived from Eqs. (3) to (5).

6 $$\frac{dm\left( t \right)}{{dt}} = \alpha \left[ {1 - \exp \left( { - \frac{t + \varepsilon }{\beta }} \right)} \right]\exp \left[ {\gamma m\left( t \right)} \right]$$

By solving indefinite integral on both sides of Eq. (6), we can obtain:

7 $$\int {\exp \left[ { - \gamma m\left( t \right)} \right]} dm\left( t \right) = \int {\alpha \left[ {1 - \exp \left( { - \frac{t + \varepsilon }{\beta }} \right)} \right]dt}$$

By solving Eq. (7), we can obtain:

8 $$m\left( t \right) = - \frac{1}{\gamma }\ln \left[ { - \alpha \gamma t - \alpha \beta \gamma \exp \left( { - \frac{t + \varepsilon }{\beta }} \right) - \gamma C} \right]$$

Using the initial condition $m\left( 0 \right) = 0$, we can get $C = - \frac{1}{\gamma } - \alpha \beta \exp \left( { - \frac{\varepsilon }{\beta }} \right)$. Therefore

9 $$m\left( t \right) = - \frac{1}{\gamma }\ln \left[ {1 - \alpha \gamma t - \alpha \beta \gamma \exp \left( { - \frac{t + \varepsilon }{\beta }} \right) + \alpha \beta \gamma \exp \left( { - \frac{\varepsilon }{\beta }} \right)} \right]$$

By solving the first-order derivatives of Eq. (9), the modified failure intensity function can be formulated as follows:

10 $$\lambda \left( t \right) = \frac{dm\left( t \right)}{{dt}} = \frac{{\alpha - \alpha \exp \left( { - \frac{t + \varepsilon }{\beta }} \right)}}{{1 - \alpha \gamma t - \alpha \beta \gamma \exp \left( { - \frac{t + \varepsilon }{\beta }} \right) + \alpha \beta \gamma \exp \left( { - \frac{\varepsilon }{\beta }} \right)}}$$

### Model parameter estimation

#### Point estimation of model parameters and reliability indices

MLE is used to estimate the parameters of reliability model. Let $t_{1} ,t_{2} , \ldots ,t_{n}$ denote the failure time of a single WT within the failure observation interval $\left[ {0,T} \right]$. When $t_{n} = T$, data is failure truncated. When $t_{n} < T$, data is time truncated. The conditional distribution function is defined to obtain the ML estimators of the model parameters.

11 $$F\left( {t_{i} \left| {t_{i - 1} } \right.} \right) = P\left( {t \le t_{i} \left| {t > t_{i - 1} } \right.} \right) = \frac{{F\left( {t_{i} } \right) - F\left( {t_{i - 1} } \right)}}{{R\left( {t_{i - 1} } \right)}} = 1 - \frac{{R\left( {t_{i} } \right)}}{{R\left( {t_{i - 1} } \right)}}$$

Before the *i*-th failure, the probability density function of the conditional reliability of WT is:

12 $$\begin{aligned} R\left( {t_{i} \left| {t_{i - 1} } \right.} \right)& = 1 - F\left( {t_{i} \left| {t_{i - 1} } \right.} \right) = \exp \left( { - \int_{{t_{i - 1} }}^{{t_{i} }} {\lambda \left( t \right)dt} } \right) \\ &= \exp \left\{ { - \alpha \exp \left[ {\left( {i - 1} \right)\gamma } \right]\left[ {t_{i} - t_{i - 1} + \beta \exp \left( { - \frac{{t_{i} + \varepsilon }}{\beta }} \right) - \beta \exp \left( { - \frac{{t_{i - 1} + \varepsilon }}{\beta }} \right)} \right]} \right\} \end{aligned}$$

The conditional probability density function for the *i-*th time of failure *t*_*i*_ is:

13 $$\begin{aligned} f\left( {t_{i} \left| {t_{i - 1} } \right.} \right) & = \alpha \exp \left[ {\left( {i - 1} \right)\gamma } \right]\left[ {1 - \exp \left( { - \frac{{t_{i} + \varepsilon }}{\beta }} \right)} \right] \\ &\quad \cdot \exp \left\{ { - \alpha \exp \left[ {\left( {i - 1} \right)\gamma } \right]\left[ {t_{i} - t_{i - 1} + \beta \exp \left( { - \frac{{t_{i} + \varepsilon }}{\beta }} \right) - \beta \exp \left( { - \frac{{t_{i - 1} + \varepsilon }}{\beta }} \right)} \right]} \right\} \end{aligned}$$

If the failure time data is time truncated, the likelihood function is:

14 $$L\left( {\alpha ,\beta ,\gamma ,\varepsilon \left| {t_{1} ,t_{2} , \ldots ,t_{n} ,T} \right.} \right) = \prod\limits_{i = 1}^{n} {f\left( {t_{i} \left| {t_{i - 1} } \right.} \right)} \cdot R\left( {T\left| {t_{n} } \right.} \right)$$

where *T* represents the end time of the test or observation. If the failure time data is failure truncated, $T = t_{n}$ and $R\left( {T\left| {t_{n} } \right.} \right) = 1$.

When failure time data for a single WT is time truncated, its likelihood function is:

15 $$\begin{aligned} l & = \prod\limits_{i = 1}^{n} {f\left( {t_{i} \left| {t_{i - 1} } \right.} \right)} R\left( {T\left| {t_{n} } \right.} \right) \\ & = \prod\limits_{i = 1}^{n} {\alpha \exp \left[ {\left( {i - 1} \right)\gamma } \right]\left[ {1 - \exp \left( { - \frac{{t_{i} + \varepsilon }}{\beta }} \right)} \right]} \\ & \quad \cdot \exp \left\{ { - \alpha \exp \left[ {\left( {i - 1} \right)\gamma } \right]\left[ {t_{i} - t_{i - 1} + \beta \exp \left( { - \frac{{t_{i} + \varepsilon }}{\beta }} \right) - \beta \exp \left( { - \frac{{t_{i - 1} + \varepsilon }}{\beta }} \right)} \right]} \right\} \\ & \quad \cdot \exp \left\{ { - \int_{{t_{n} }}^{T} {\alpha \left[ {1 - \exp \left( { - \frac{t + \varepsilon }{\beta }} \right)} \right] \cdot \exp \left[ {\gamma N\left( t \right)dt} \right]} } \right\} \\ & = \alpha^{n} \cdot \exp \left[ {\frac{{n\left( {n - 1} \right)}}{2}\gamma } \right]\prod\limits_{i = 1}^{n} {\left[ {1 - \exp \left( { - \frac{{t_{i} + \varepsilon }}{\beta }} \right)} \right]} \\ & \quad \cdot \exp \left\{ { - \alpha \exp \left[ {\left( {i - 1} \right)\gamma } \right]\left[ {t_{i} - t_{i - 1} + \beta \exp \left( { - \frac{{t_{i} + \varepsilon }}{\beta }} \right) - \beta \exp \left( { - \frac{{t_{i - 1} + \varepsilon }}{\beta }} \right)} \right]} \right\} \\ & \quad \cdot \exp \left\{ { - \alpha \exp \left( {n\gamma } \right) \cdot \left[ {T - t_{n} + \beta \exp \left( { - \frac{T + \varepsilon }{\beta }} \right) - \beta \exp \left( { - \frac{{t_{n} + \varepsilon }}{\beta }} \right)} \right]} \right\} \\ \end{aligned}$$

Taking the natural log on both sides of Eq. (15)

16 $$\begin{aligned} \ln l & = n\ln \alpha + \frac{{n\left( {n - 1} \right)}}{2}\gamma + \sum\limits_{i = 1}^{n} {\left\{ {\ln \left[ {1 - \exp \left( { - \frac{{t_{i} + \varepsilon }}{\beta }} \right)} \right]} \right.} \\&\quad- \alpha \sum\limits_{i = 1}^{n} {\exp \left[ {\left( {i - 1} \right)\gamma } \right]\left[ {t_{i} - t_{i - 1} + \beta \exp \left( { - \frac{{t_{i} + \varepsilon }}{\beta }} \right) - \beta \exp \left( { - \frac{{t_{i - 1} + \varepsilon }}{\beta }} \right)} \right]} \\ & \quad \left. { - \alpha \exp \left( {n\gamma } \right)\left[ {T - t_{n} + \beta \exp \left( { - \frac{T + \varepsilon }{\beta }} \right) - \beta \exp \left( { - \frac{{t_{n} + \varepsilon }}{\beta }} \right)} \right]} \right\} \\ \end{aligned}$$

When failure time data is failure truncated, its likelihood function is:

17 $$\begin{aligned} L & = \prod\limits_{i = 1}^{n} {\alpha \exp \left[ {\left( {i - 1} \right)\gamma } \right]\left[ {1 - \exp \left( { - \frac{{t_{i} + \varepsilon }}{\beta }} \right)} \right]} \\ &\quad\cdot \exp \left\{ { - \alpha \exp \left[ {\left( {i - 1} \right)\gamma } \right]\left[ {t_{i} - t_{i - 1} + \beta \exp \left( { - \frac{{t_{i} + \varepsilon }}{\beta }} \right) - \beta \exp \left( { - \frac{{t_{i - 1} + \varepsilon }}{\beta }} \right)} \right]} \right\} \\ & = \alpha^{n} \cdot \exp \left[ {\frac{{n\left( {n - 1} \right)}}{2}\gamma } \right]\prod\limits_{i = 1}^{n} {\left[ {1 - \exp \left( { - \frac{{t_{i} + \varepsilon }}{\beta }} \right)} \right]} \\ &\quad\cdot \exp \left\{ { - \alpha \exp \left[ {\left( {i - 1} \right)\gamma } \right]\left[ {t_{i} - t_{i - 1} + \beta \exp \left( { - \frac{{t_{i} + \varepsilon }}{\beta }} \right) - \beta \exp \left( { - \frac{{t_{i - 1} + \varepsilon }}{\beta }} \right)} \right]} \right\} \\ \end{aligned}$$

Taking the natural log on both sides of Eq. (17)

18 $$\ln L = n\ln \alpha + \frac{{n\left( {n - 1} \right)}}{2}\gamma + \sum\limits_{i = 1}^{n} {\left\{ {\ln \left[ {1 - \exp \left( { - \frac{{t_{i} + \varepsilon }}{\beta }} \right)} \right]} \right.} \left. { - \alpha \sum\limits_{i = 1}^{n} {\exp \left[ {\left( {i - 1} \right)\gamma } \right]\left[ {t_{i} - t_{i - 1} + \beta \exp \left( { - \frac{{t_{i} + \varepsilon }}{\beta }} \right) - \beta \exp \left( { - \frac{{t_{i - 1} + \varepsilon }}{\beta }} \right)} \right]} } \right\}$$

Taking the first partial derivatives of Eq. (18) with respect to the model parameters $\alpha$, $\beta$, $\gamma$, $\varepsilon$, respectively.

19 $$\frac{\partial \ln L}{{\partial \alpha }} = \frac{n}{\alpha } - \sum\limits_{i = 1}^{n} {\exp \left[ {\left( {i - 1} \right)\gamma } \right] \cdot \left[ {t_{i} - t_{i - 1} + \beta \exp \left( { - \frac{{t_{i} + \varepsilon }}{\beta }} \right) - \beta \exp \left( { - \frac{{t_{i - 1} + \varepsilon }}{\beta }} \right)} \right]}$$

20 $$\begin{aligned} \frac{\partial \ln L}{{\partial \beta }} & = \sum\limits_{i = 1}^{n} {\frac{{\exp \left( { - \frac{{t_{i} + \varepsilon }}{\beta }} \right)\left( {t_{i} + \varepsilon } \right)}}{{\beta^{2} \left[ {\exp \left( { - \frac{{t_{i} + \varepsilon }}{\beta }} \right) - 1} \right]}}} - \alpha \sum\limits_{i = 1}^{n} {\exp \left[ {\left( {i - 1} \right)\gamma } \right]\left[ {\exp \left( { - \frac{{t_{i} + \varepsilon }}{\beta }} \right) + \frac{{t_{i} + \varepsilon }}{\beta }\exp \left( { - \frac{{t_{i} + \varepsilon }}{\beta }} \right)} \right.} \\ & \quad \left. { - \exp \left( { - \frac{{t_{i - 1} + \varepsilon }}{\beta }} \right) - \frac{{t_{i - 1} + \varepsilon }}{\beta }\exp \left( { - \frac{{t_{i - 1} + \varepsilon }}{\beta }} \right)} \right] \\ \end{aligned}$$

21 $$\frac{\partial \ln L}{{\partial \gamma }} = \frac{{n\left( {n - 1} \right)}}{2} - \alpha \sum\limits_{i = 1}^{n} {\left( {i - 1} \right)\exp \left[ {\left( {i - 1} \right)\gamma } \right]} \left[ {t_{i} - t_{i - 1} + \beta \exp \left( { - \frac{{t_{i} + \varepsilon }}{\beta }} \right) - \beta \exp \left( { - \frac{{t_{i - 1} + \varepsilon }}{\beta }} \right)} \right]$$

22 $$\frac{\partial \ln L}{{\partial \varepsilon }} = \sum\limits_{i = 1}^{n} {\frac{{ - \exp \left( { - \frac{{t_{i} + \varepsilon }}{\beta }} \right)}}{{\beta \left[ {\exp \left( { - \frac{{t_{i} + \varepsilon }}{\beta }} \right) - 1} \right]}}} - \alpha \sum\limits_{i = 1}^{n} {\exp \left[ {\left( {i - 1} \right)\gamma } \right]\left[ { - \exp \left( { - \frac{{t_{i} + \varepsilon }}{\beta }} \right) + \exp \left( { - \frac{{t_{i - 1} + \varepsilon }}{\beta }} \right)} \right]}$$

Setting Eqs. (19) to (22) equal to zero, the MLE of model parameters satisfy Eq. (23):

23 $$\left\{ \begin{gathered} \frac{\partial \ln L}{{\partial \alpha }} = 0 \hfill \\ \frac{\partial \ln L}{{\partial \beta }} = 0 \hfill \\ \frac{\partial \ln L}{{\partial \gamma }} = 0 \hfill \\ \frac{\partial \ln L}{{\partial \varepsilon }} = 0 \hfill \\ \end{gathered} \right.$$

Using the PSO algorithm to solve Eq. (23), the maximum likelihood point estimators $\hat{\alpha }$, $\hat{\beta }$, $\hat{\gamma }$, $\hat{\varepsilon }$ can be obtained. Let the Eq. (23) be $f_{1} \left( t \right) = \frac{\partial \ln L}{{\partial \alpha }}$, $f_{2} \left( t \right) = \frac{\partial \ln L}{{\partial \beta }}$, $f_{3} \left( t \right) = \frac{\partial \ln L}{{\partial \gamma }}$, $f_{4} \left( t \right) = \frac{\partial \ln L}{{\partial \varepsilon }}$. Rewrite Eq. (23) as:

24 $$\left\{ {\begin{array}{*{20}l} {f_{1} \left( t \right) = \frac{{\partial \ln L}}{{\partial \alpha }}} \hfill \\ {f_{2} \left( t \right) = \frac{{\partial \ln L}}{{\partial \beta }}} \hfill \\ {f_{3} \left( t \right) = \frac{{\partial \ln L}}{{\partial \gamma }}} \hfill \\ {f_{4} \left( t \right) = \frac{{\partial \ln L}}{{\partial \varepsilon }}} \hfill \\ {f\left( t \right) = f_{1}^{2} \left( t \right) + f_{2}^{2} \left( t \right) + f_{3}^{2} \left( t \right) + f_{4}^{2} \left( t \right)} \hfill \\ \end{array} } \right.$$

Solving Eq. (24) is equivalent to solving the following optimization problem:

25 $$\min \left\{ {\max f\left( t \right)} \right\}$$

Since the objective function consists of four transcendental equations, the search for the optimal solution begins by considering the maximum value $\max f\left( t \right)$ of any possible objective function solution. On this basis, the solution space is searched to minimise the solution space's maximum value $\min \left\{ {\max f\left( t \right)} \right\}$. Consequently, the fitness of the corresponding model parameters is the highest, and the solution is close to the actual value. If Eq. (23) has a solution, then the minimum value of Eq. (25)'s objective function is 0. In other words, the solution of Eq. (24) is more precise if the minimum value of the objective function in Eq. (25) is closer to 0.

The maximum likelihood point estimators of the WT reliability indices can be expressed as follows:

1. The point estimator of the instantaneous failure intensity function can be expressed as:
  26 $$\hat{\lambda }\left( t \right) = \frac{{\hat{\alpha } - \hat{\alpha }\exp \left( { - \frac{{t + \hat{\varepsilon }}}{{\hat{\beta }}}} \right)}}{{1 - \hat{\alpha }\hat{\gamma }t - \hat{\alpha }\hat{\beta }\hat{\gamma }\exp \left( { - \frac{{t + \hat{\varepsilon }}}{{\hat{\beta }}}} \right) + \hat{\alpha }\hat{\beta }\hat{\gamma }\exp \left( { - \frac{{\hat{\varepsilon }}}{{\hat{\beta }}}} \right)}}$$
2. The instantaneous mean time between failure (IMTBF) can be expressed as:
  27 $$u\left( t \right) = \frac{1}{\lambda \left( t \right)}$$
  Its point estimator is:
  28 $$\hat{u}\left( t \right) = \frac{{1 - \hat{\alpha }\hat{\gamma }t - \hat{\alpha }\hat{\beta }\hat{\gamma }\exp \left( { - \frac{{t + \hat{\varepsilon }}}{{\hat{\beta }}}} \right) + \hat{\alpha }\hat{\beta }\hat{\gamma }\exp \left( { - \frac{{\hat{\varepsilon }}}{{\hat{\beta }}}} \right)}}{{\hat{\alpha } - \hat{\alpha }\exp \left( { - \frac{{t + \hat{\varepsilon }}}{{\hat{\beta }}}} \right)}}$$
3. The average cumulative point estimator of failures can be expressed as:
  29 $$\hat{m}\left( t \right) = - \frac{1}{{\hat{\gamma }}}\ln \left[ {1 - \hat{\alpha }\hat{\gamma }t - \hat{\alpha }\hat{\beta }\hat{\gamma }\exp \left( { - \frac{{t + \hat{\varepsilon }}}{{\hat{\beta }}}} \right) + \hat{\alpha }\hat{\beta }\hat{\gamma }\exp \left( { - \frac{{\hat{\varepsilon }}}{{\hat{\beta }}}} \right)} \right]$$
4. The cumulative mean time between failure (CMTBF) can be expressed as:
  30 $$u_{c} \left( t \right) = \frac{t}{m\left( t \right)}$$
  Its point estimator is:
  31 $$\hat{u}_{c} \left( t \right) = - \frac{{\hat{\gamma }t}}{{\ln \left[ {1 - \hat{\alpha }\hat{\gamma }t - \hat{\alpha }\hat{\beta }\hat{\gamma }\exp \left( { - \frac{{t + \hat{\varepsilon }}}{{\hat{\beta }}}} \right) + \hat{\alpha }\hat{\beta }\hat{\gamma }\exp \left( { - \frac{{\hat{\varepsilon }}}{{\hat{\beta }}}} \right)} \right]}}$$
5. The cumulative failure intensity function can be expressed as:
  32 $$\lambda_{c} \left( t \right) = \frac{1}{{u_{c} \left( t \right)}}$$
  Its point estimator is:
  33 $$\hat{\lambda }_{c} \left( t \right) = - \frac{{\ln \left[ {1 - \hat{\alpha }\hat{\gamma }t - \hat{\alpha }\hat{\beta }\hat{\gamma }\exp \left( { - \frac{{t + \hat{\varepsilon }}}{{\hat{\beta }}}} \right) + \hat{\alpha }\hat{\beta }\hat{\gamma }\exp \left( { - \frac{{\hat{\varepsilon }}}{{\hat{\beta }}}} \right)} \right]}}{{\hat{\gamma }t}}$$
6. The reliability function can be expressed as:
  34 $$R\left( t \right) = \exp - \int_{0}^{t} {\lambda \left( t \right)dt}$$
  Its point estimator is:
  35 $$\hat{R}\left( t \right) = \left[ {1 + \hat{\alpha }\hat{\beta }\hat{\gamma }\exp \left( { - \frac{{\hat{\varepsilon }}}{{\hat{\beta }}}} \right) - \hat{\alpha }\hat{\gamma }t - \hat{\alpha }\hat{\beta }\hat{\gamma }\exp \left( { - \frac{{t + \hat{\varepsilon }}}{{\hat{\beta }}}} \right)} \right]^{{\frac{1}{{\hat{\gamma }}}}}$$

#### Interval estimation of model parameters and reliability indices

The Delta method^26,27^ is used to estimate the confidence interval of model parameters and reliability indices. The Delta method uses the principle of Taylor expansion to obtain the mean and variance of approximate complex statistics. The Delta method for interval estimation only needs to calculate the model parameters, and the variance and covariance of the parameters. Then the approximate standard error of the smooth function of the parameters can be obtained. Asymptotic lognormal distribution of maximum likelihood estimators is used to estimate confidence intervals for model parameter values.

36 $$\left[ {\hat{\theta }_{L} ,\hat{\theta }_{U} } \right] = \hat{\theta }\exp \left( { \pm \frac{{Z_{{\frac{\mu }{2}}} \hat{\sigma }_{{\hat{\theta }}} }}{{\hat{\theta }}}} \right)$$

where $\hat{\theta }$ represents the estimated model parameters or reliability indices and $Z_{\mu /2}$ is the quantile of the standard normal distribution with a level of confidence $100\left( {1 - \mu } \right)\%$.The random interval $\left[ {\hat{\theta }_{L} ,\hat{\theta }_{U} } \right]$ is the confidence interval of $\theta$ at the confidence level $1 - \mu$. $\hat{\theta }_{L}$ and $\hat{\theta }_{U}$ are the lower and upper limits of the confidence interval, respectively. $\hat{\sigma }_{{\hat{\theta }}}$ (to be estimated) is the standard deviation of model parameters or reliability indices, $\hat{\sigma }_{{\hat{\theta }}} = \sqrt {{\text{var}} \left( {\hat{\theta }} \right)}$. ${\text{var}} \left( {\hat{\theta }} \right)$ is the variance of model parameters or reliability indices. The confidence interval can be obtained by calculating the variance of model parameters or reliability indices.

According to the inverse Fisher information matrix method, the model parameter variance (var) and covariance (cov) are as follows:

37 $$\left[ {\begin{array}{*{20}c} {{\text{var}} \left( {\hat{\alpha }} \right)} & {{\text{cov}} \left( {\hat{\alpha },\hat{\beta }} \right)} & {{\text{cov}} \left( {\hat{\alpha },\hat{\gamma }} \right)} & {{\text{cov}} \left( {\hat{\alpha },\hat{\varepsilon }} \right)} \\ {{\text{cov}} \left( {\hat{\alpha },\hat{\beta }} \right)} & {{\text{var}} \left( {\hat{\beta }} \right)} & {{\text{cov}} \left( {\hat{\beta },\hat{\gamma }} \right)} & {{\text{cov}} \left( {\hat{\beta },\hat{\varepsilon }} \right)} \\ {{\text{cov}} \left( {\hat{\alpha },\hat{\gamma }} \right)} & {{\text{cov}} \left( {\hat{\beta },\hat{\gamma }} \right)} & {{\text{var}} \left( {\hat{\gamma }} \right)} & {{\text{cov}} \left( {\hat{\gamma },\hat{\varepsilon }} \right)} \\ {{\text{cov}} \left( {\hat{\alpha },\hat{\varepsilon }} \right)} & {{\text{cov}} \left( {\hat{\beta },\hat{\varepsilon }} \right)} & {{\text{cov}} \left( {\hat{\gamma },\hat{\varepsilon }} \right)} & {{\text{var}} \left( {\hat{\varepsilon }} \right)} \\ \end{array} } \right] = \left[ {\begin{array}{*{20}c} { - \frac{{\partial^{2} \ln L}}{{\partial \hat{\alpha }^{2} }}} & { - \frac{{\partial^{2} \ln L}}{{\partial \hat{\alpha }\partial \hat{\beta }}}} & { - \frac{{\partial^{2} \ln L}}{{\partial \hat{\alpha }\partial \hat{\gamma }}}} & { - \frac{{\partial^{2} \ln L}}{{\partial \hat{\alpha }\partial \hat{\varepsilon }}}} \\ { - \frac{{\partial^{2} \ln L}}{{\partial \hat{\alpha }\partial \hat{\beta }}}} & { - \frac{{\partial^{2} \ln L}}{{\partial \hat{\beta }^{2} }}} & { - \frac{{\partial^{2} \ln L}}{{\partial \hat{\beta }\partial \hat{\gamma }}}} & { - \frac{{\partial^{2} \ln L}}{{\partial \hat{\beta }\partial \hat{\varepsilon }}}} \\ { - \frac{{\partial^{2} \ln L}}{{\partial \hat{\alpha }\partial \hat{\gamma }}}} & { - \frac{{\partial^{2} \ln L}}{{\partial \hat{\beta }\partial \hat{\gamma }}}} & { - \frac{{\partial^{2} \ln L}}{{\partial \hat{\gamma }^{2} }}} & { - \frac{{\partial^{2} \ln L}}{{\partial \hat{\gamma }\partial \hat{\varepsilon }}}} \\ { - \frac{{\partial^{2} \ln L}}{{\partial \hat{\alpha }\partial \hat{\varepsilon }}}} & { - \frac{{\partial^{2} \ln L}}{{\partial \hat{\beta }\partial \hat{\varepsilon }}}} & { - \frac{{\partial^{2} \ln L}}{{\partial \hat{\gamma }\partial \hat{\varepsilon }}}} & { - \frac{{\partial^{2} \ln L}}{{\partial \hat{\varepsilon }^{2} }}} \\ \end{array} } \right]^{ - 1}$$

The entries in Eq. (37) are derived by taking the second partial derivative of each parameter in Eq. (18). The specific derivation process is illustrated in ESM Appendix Eqs. (A1) to (A10). Substitute the values of ESM Appendix Eqs. (A1) to (A10) into the right side of Eq. (37), and then solve the inverse matrix on the right side to obtain ${\text{var}} \left( {\hat{\alpha }} \right)$, ${\text{var}} \left( {\hat{\beta }} \right)$, ${\text{var}} \left( {\hat{\gamma }} \right)$ and ${\text{var}} \left( {\hat{\varepsilon }} \right)$. Substituting them into Eq. (36) to estimate the confidence interval for the parameters, respectively.

Then let $\varphi$ represent the reliability indices $\lambda \left( t \right)$, $u\left( t \right)$, $m\left( t \right)$, $\lambda_{c} \left( t \right)$ $u_{c} \left( t \right)$ and $R\left( t \right)$, respectively. The variance of $\varphi$ is:

38 $$\begin{aligned} {\text{var}} \left( {\hat{\varphi }} \right) & = \left( {\frac{\partial \varphi }{{\partial \alpha }}} \right)^{2} {\text{var}} \left( {\hat{\alpha }} \right) + \left( {\frac{\partial \varphi }{{\partial \beta }}} \right)^{2} {\text{var}} \left( {\hat{\beta }} \right) + \left( {\frac{\partial \varphi }{{\partial \gamma }}} \right)^{2} {\text{var}} \left( {\hat{\gamma }} \right) + \left( {\frac{\partial \varphi }{{\partial \varepsilon }}} \right)^{2} {\text{var}} \left( {\hat{\varepsilon }} \right) \\ & \quad + 2\left( {\frac{\partial \varphi }{{\partial \alpha }}} \right)\left( {\frac{\partial \varphi }{{\partial \beta }}} \right){\text{cov}} \left( {\hat{\alpha },\hat{\beta }} \right) + 2\left( {\frac{\partial \varphi }{{\partial \alpha }}} \right)\left( {\frac{\partial \varphi }{{\partial \gamma }}} \right){\text{cov}} \left( {\hat{\alpha },\hat{\gamma }} \right) + 2\left( {\frac{\partial \varphi }{{\partial \alpha }}} \right)\left( {\frac{\partial \varphi }{{\partial \varepsilon }}} \right){\text{cov}} \left( {\hat{\alpha },\hat{\varepsilon }} \right) \\ & \quad + 2\left( {\frac{\partial \varphi }{{\partial \beta }}} \right)\left( {\frac{\partial \varphi }{{\partial \gamma }}} \right){\text{cov}} \left( {\hat{\beta },\hat{\gamma }} \right) + 2\left( {\frac{\partial \varphi }{{\partial \beta }}} \right)\left( {\frac{\partial \varphi }{{\partial \varepsilon }}} \right){\text{cov}} \left( {\hat{\beta },\hat{\varepsilon }} \right) + 2\left( {\frac{\partial \varphi }{{\partial \gamma }}} \right)\left( {\frac{\partial \varphi }{{\partial \varepsilon }}} \right){\text{cov}} \left( {\hat{\gamma },\hat{\varepsilon }} \right) \\ \end{aligned}$$

The reliability indices $\lambda \left( t \right)$, $u\left( t \right)$, $m\left( t \right)$, $\lambda_{c} \left( t \right)$, $u_{c} \left( t \right)$ and $R\left( t \right)$ are used to replace $\varphi$. To obtain the corresponding partial derivative from Eq. (38), the specific derivation process is illustrated in ESM Appendix Eqs. (A11) to (A34). The variance (var) and covariance (cov) determined by Eq. (37) are then substituted into Eq. (38). Finally, substituting them into Eq. (36) yields the confidence interval for the reliability indices, respectively.

## Application

The failure time data of 12 WTs from Dabancheng wind farm in Xinjiang Uygur Autonomous Region of China are used to illustrate the proposed model and related estimation. The WTs have a 20-year design life and have been in service for 10 years. Through the collection and sorting of alarm logs, repair records, and other data, as well as field investigation and verification, the failure time data of 12 WTs for the three years from 00:00 on January 1, 2019, to 23:59 on December 31, 2021, was compiled. The total duration of observation is *T* = 26280 h. The failure time data is provided in ESM Appendix Table A1.

### Failure time data trend test

To test the trend of failure time data using the TTT (Total time on test) graph test method^4^, Let *n* represent the total number of WT failures, *S*_0_ = 0, and compute the TTT value S_*i*_. Regularize TTT value S_*i*_, i.e., $u_{i} = S_{i} /S_{n}$.

39 $$S_{i} = nt_{i} + \left( {n - 1} \right)\left( {t_{2} - t_{1} } \right) + \cdots + \left( {n - i + 1} \right)\left( {t_{i} - t_{i - 1} } \right),\quad i = 1,2, \ldots ,n$$

TTT graph is depicted with *i/n* as the abscissa and $u_{i}$ ordinate, and the results are shown in ESM Appendix Fig. A1. The ratio of the *i*-th number of failures to the total number of failures is denoted by *i/n*.

It is shown in ESM Appendix Fig. A1 that except for individual failure points of individual WT in the early period, other scattered points are distributed above the diagonal of the unit square. This indicated that the failure intensity of the WT is increasing and they are in the wear-out period. Accordingly, the proposed model can be used for reliability modeling of WT.

### Model parameters estimation

The failure time data in ESM Appendix Table A1 was substituted into the proposed model, and the PSO solved the maximum likelihood estimation of model parameters for 12 WTs. Using the inverse Fisher information matrix method, the 95% confidence interval for model parameters was calculated. The model parameters estimation result is shown in Table 1.

**Table 1:** Point estimation and interval estimation of model parameters.

WTs	Model parameter	Point estimation	Confidence interval with 95% confidence
1#	$\hat{\alpha }$ (× 10^−3^)	8.1207673	[8.1207673, 8.1207673]
	$\hat{\beta }$	25,744.80	[25,744.79, 25,744.81]
	$\hat{\gamma }$ (× 10^−2^)	− 3.905	[− 5.797. − 2.630]
	$\hat{\varepsilon }$	1406.80	[1406.79, 1406.81]
2#	$\hat{\alpha }$ (× 10^−3^)	4.1467515	[4.1467515, 4.1467515]
	$\hat{\beta }$	25,704.00	[25,703.99, 24,704.01]
	$\hat{\gamma }$ (× 10^−2^)	− 1.840	[− 4.434, − 0.764]
	$\hat{\varepsilon }$	5146.00	[5145.99, 5146.02]
3#	$\hat{\alpha }$ (× 10^−3^)	4.9857479	[4.9857447, 4.9857511]
	$\hat{\beta }$	11,832.82	[11,831.33, 11,834.31]
	$\hat{\gamma }$ (× 10^−2^)	− 8.874	[− 12.765, − 6.168]
	$\hat{\varepsilon }$	340.00	[337.28, 342.74]
4#	$\hat{\alpha }$ (× 10^−3^)	1.361145	[1.3611443, 1.3611458]
	$\hat{\beta }$	5268.23	[5267.06, 5269.40]
	$\hat{\gamma }$ (× 10^−2^)	− 1.783	[− 10.104, − 0.315]
	$\hat{\varepsilon }$	1559.59	[1554.06, 1565.13]
5#	$\hat{\alpha }$ (× 10^−3^)	3.4632139	[3.4632139, 3.4632139]
	$\hat{\beta }$	21,693.16	[21,693.14, 21,693.18]
	$\hat{\gamma }$ (× 10^−2^)	− 0.652	[− 7.242, − 0.059]
	$\hat{\varepsilon }$	1738.99	[1738.97, 1739.01]
6#	$\hat{\alpha }$ (× 10^−3^)	5.869942	[5.8699415, 5.8699424]
	$\hat{\beta }$	24,300.47	[24,299.69, 24,301.25]
	$\hat{\gamma }$ (× 10^−2^)	− 5.524	[− 9.008, − 3.388]
	$\hat{\varepsilon }$	149.50	[148.74, 150.27]
7#	$\hat{\alpha }$ (× 10^−3^)	2.2798169	[2.2797713, 2.2798626]
	$\hat{\beta }$	15,068.75	[15,058.71, 15,078.80]
	$\hat{\gamma }$ (× 10^−2^)	− 4.838	[− 11.727, − 1.996]
	$\hat{\varepsilon }$	613.12	[595.96, 630.78]
8#	$\hat{\alpha }$ (× 10^−3^)	8.788633	[8.788633, 8.788633]
	$\hat{\beta }$	25,040.69	[25,040.61, 25,040.77]
	$\hat{\gamma }$ (× 10^−2^)	− 6.242	[− 8.845, − 4.405]
	$\hat{\varepsilon }$	414.00	[413.93, 414.07]
9#	$\hat{\alpha }$ (× 10^−3^)	11.402081	[11.402005, 11.402157]
	$\hat{\beta }$	24,945.80	[24,936.43, 24,955.18]
	$\hat{\gamma }$ (× 10^−2^)	− 17.396	[− 22.926, − 13.200]
	$\hat{\varepsilon }$	584.00	[574.98, 593.16]
10#	$\hat{\alpha }$ (× 10^−3^)	37.134564	[37.134564, 37.134564]
	$\hat{\beta }$	25,836.48	[25,836.45, 25,836.50]
	$\hat{\gamma }$ (× 10^−2^)	− 15.031	[− 17.595, − 12.841]
	$\hat{\varepsilon }$	768.00	[767.98, 768.02]
11#	$\hat{\alpha }$ (× 10^−3^)	22.384214	[22.384214, 22.384214]
	$\hat{\beta }$	24,945.30	[24,945.299, 24,945.301]
	$\hat{\gamma }$ (× 10^−2^)	− 8.090	[− 9.843, − 6.648]
	$\hat{\varepsilon }$	655.30	[655.299, 655.301]
12#	$\hat{\alpha }$ (× 10^−3^)	3.5584901	[3.55849, 3.5584901]
	$\hat{\beta }$	24,096.00	[24,095.67, 24,096.33]
	$\hat{\gamma }$ (× 10^−2^)	− 1.734	[− 6.330, -0.475]
	$\hat{\varepsilon }$	3376.50	[3376.14, 3376.86]

**Figure 1:**
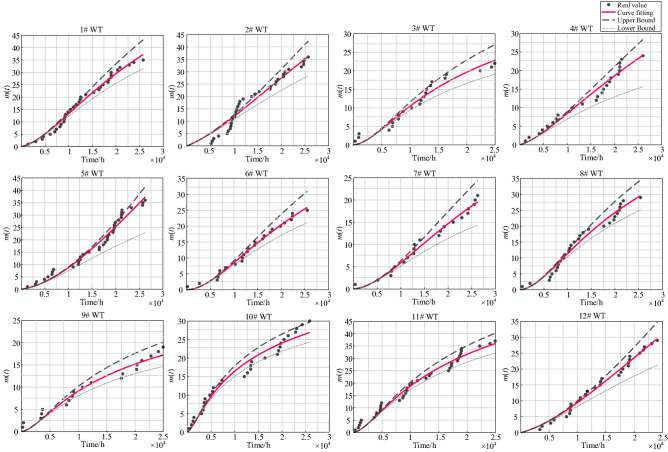
Maximum likelihood estimation fitting curves of the cumulative number of failures.

Figure 1 shows the fitting curves of the estimation of the cumulative number of failures $m\left( t \right)$ and its 95% confidence interval for 12 WTs. It can be seen from Fig. 1 that the estimated cumulative failure number curves fit well with the failure time data.

**Figure 2:**
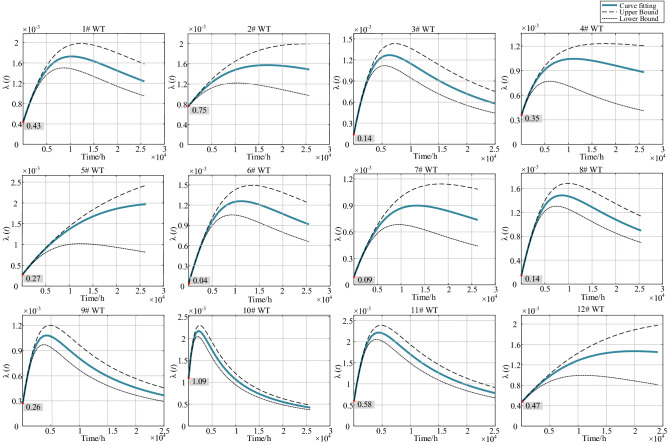
Results of fitting the instantaneous failure intensity function.

The fitting results of the instantaneous failure intensity function and its 95% confidence interval for 12 WTs are shown in Fig. 2. As is illustrated in Fig. 2, the failure intensity of all WTs at the initial time was not zero. As the random failure period preceded the actual wear-out period, and the failure intensity of the random failure period was not zero. The failure intensity of WTs during the wear-out period is increasing, but not indefinitely; rather, it approaches a constant. This indicates that the reliability model presented in this paper, using 3-BIP as the benchmark failure intensity function of LPIM, accorded with the engineering practice well.

### Goodness of fit test

In order to show the fitting capabilities of proposed reliability model more intuitively, it is necessary to evaluate the goodness-of-fit (GOF) of the proposed model. Different models have different responses to failure data. The best model has a high degree of fitting, few parameters, and a simple form^28–30^. Based on the concept of entropy, the Akaike information criterion (AIC)^4,28,30^ can balance the complexity of the estimation model and the quality of data fitting. The greater the sample size and the smaller the AIC value, the better the model-fitting effect. However, when the number of samples is excessively high, overfitting is common. On this basis, the Bayesian information criterion (BIC)^28,31^ with a relatively larger penalty term is introduced, which can effectively prevent the issue of excessive model complexity resulting from excessive model accuracy. The AIC and BIC make use of the likelihood estimation property and hold that the best model should have the smallest AIC and BIC values. They are common methods for model selection. Their expressions are described as:

40 $${\text{AIC}} = - 2\max \ln L + 2m$$

41 $${\text{BIC}} = - 2\max \ln L + m\ln n$$

where *m* is the number of model parameters, *n* is the number of failure time data, and $\max \ln L$ is the maximum log-likelihood function of failure time data.

To further verify that the proposed model is the best model for describing the WT failure process, the correlative coefficient *R* between the actual value and the fitting value of the cumulative number of failures is used to evaluate the GOF of different models. The *R* is defined as:

42 $$R = 1 - \sqrt {\sum\limits_{i = 1}^{N} {\left( {N_{i} - \hat{M}_{i} } \right)^{2} } \bigg/\sum\limits_{i = 1}^{N} {N_{i}^{2} } }$$

where $N_{i}$ is the actual cumulative number of failures at the time *i*; $\hat{M}_{i}$ represents the fitting value of the cumulative number of failures. We know that the closer the value of *R* is to one, the higher the fitting accuracy of the model.

Table 2 shows that the AIC, BIC and* R* calculation results of 12 WTs, respectively. Table 2 notes that the average values of AIC, BIC and* R* of 12 WTs calculated based on the proposed model are 3339.83, 344.45 and 0.9173, respectively. Compared with LPIM and 3-BIP, the proposed reliability model has higher fitting accuracy.

**Table 2:** Comparison of GOF results of different reliability model.

WTs	LPIM			3-BIP			Proposed model		
	AIC	BIC	R	AIC	BIC	R	AIC	BIC	R
1#	409.90	420.57	0.8612	412.64	423.31	0.8949	393.16	399.26	0.9390
2#	432.42	443.17	0.8873	435.12	445.87	0.7697	419.77	425.99	0.8861
3#	313.66	322.93	0.8193	304.42	313.69	0.8354	277.38	281.56	0.8923
4#	342.30	351.83	0.9001	347.16	356.69	0.9010	327.11	331.65	0.9064
5#	427.76	438.51	0.8841	442.04	452.79	0.8761	420.95	427.17	0.9150
6#	313.94	323.60	0.8793	322.52	332.18	0.8904	301.47	306.19	0.9487
7#	301.42	310.55	0.8805	297.78	306.91	0.9007	282.31	286.29	0.9346
8#	378.86	388.96	0.9003	384.40	394.50	0.8567	340.68	346.01	0.9182
9#	260.12	268.95	0.8671	264.24	273.07	0.8206	238.29	241.85	0.8995
10#	364.16	374.36	0.8434	367.88	378.08	0.8672	334.88	340.35	0.9043
11#	406.54	417.37	0.8076	412.16	422.99	0.9101	396.26	402.60	0.9206
12#	353.36	363.46	0.8364	360.50	370.60	0.8136	345.65	350.98	0.9425
Average value	358.70	368.69	0.8639	362.57	372.56	0.8614	339.83	344.45	0.9173

### Reliability indices estimation and analysis

The reliability of WT after the last failure is more concerned by operation and maintenance personnel. The point estimation results of model parameters from Table 1 were substituted into Eqs. (26) to (35). The point estimation result of key reliability indices at the last failure time *t* for every WT was computed. The confidence interval of reliability indices with 95% confidence were solved using the Delta method. The result is shown in Table 3.

**Table 3:** Point estimation and interval estimation of reliability indices.

WTs	Reliability indices	Point estimation	Confidence interval with 95% confidence
1#	$\lambda \left( {t = 25744.8{\text{h}} } \right)$	1.24 × 10^−3^	[0.96 × 10^−3^, 1.60 × 10^−3^]
	${\text{u}}\left( {t = 25744.8{\text{h}} } \right)$	808.30	[626.90, 1042.20]
	${\text{m}}\left( {t = 25744.8{\text{h}} } \right)$	37.22	[31.82, 43.54]
	${\text{u}}_{{\text{c}}} \left( {t = 25744.8{\text{h}} } \right)$	691.67	[591.28, 809.11]
	$\lambda_{c} \left( {t = 25744.8{\text{h}} } \right)$	1.45 × 10^−3^	[1.24 × 10^−3^, 1.69 × 10^−3^]
	$R\left( {t = 691.67{\text{h}} } \right)$	0.69290741	[0.69290607, 0.69290858]
2#	$\lambda \left( {t = 25704{\text{h}} } \right)$	1.49 × 10^−3^	[1.04 × 10^−3^, 2.13 × 10^−3^]
	$u\left( {t = 25704{\text{h}} } \right)$	671.67	[469.10, 961.72]
	$m\left( {t = 25704{\text{h}} } \right)$	36.19	[29.66, 44.17]
	$u_{c} \left( {t = 25704{\text{h}} } \right)$	710.17	[581.87, 866.75]
	$\lambda_{c} \left( {t = 25704{\text{h}} } \right)$	1.41 × 10^−3^	[1.154 × 10^−3^, 1.72 × 10^−3^]
	$R\left( {t = 710.17{\text{h}} } \right)$	0.56872225	[0.56871939, 0.56872546]
3#	$\lambda \left( {t = 24945.5{\text{h}} } \right)$	0.58 × 10^−3^	[0.45 × 10^−3^, 0.76 × 10^−3^]
	$u\left( {t = 24945.5{\text{h}} } \right)$	1720.90	[1320.49, 2242.71]
	$m\left( {t = 24945.5{\text{h}} } \right)$	22.81	[19.16, 27.15]
	$u_{c} \left( {t = 24945.5{\text{h}} } \right)$	1093.74	[918.78, 1302.01]
	$\lambda_{c} \left( {t = 24945.5{\text{h}} } \right)$	0.91 × 10^−3^	[0.77 × 10^−3^, 1.09 × 10^−3^]
	$R\left( {t = 1093.74{\text{h}} } \right)$	0.68024261	[0.68024033, 0.68024433]
4#	$\lambda \left( {t = 26037.5{\text{h}} } \right)$	0.88 × 10^−3^	[0.53 × 10^−3^, 1.46 × 10^−3^]
	$u\left( {t = 26037.5{\text{h}} } \right)$	1135.68	[682.69, 1889.27]
	$m\left( {t = 26037.5{\text{h}} } \right)$	24.12	[18.37, 31.69]
	$u_{c} \left( {t = 26037.5{\text{h}} } \right)$	1079.31	[821.73, 1417.64]
	$\lambda_{c} \left( {t = 26037.5{\text{h}} } \right)$	0.93 × 10^−3^	[0.71 × 10^−3^, 1.22 × 10^−3^]
	$R\left( {t = 1079.31{\text{h}} } \right)$	0.61937203	[0.61936998, 0.61937565]
5#	$\lambda \left( {t = 26236.5{\text{h}} } \right)$	1.97 × 10^−3^	[1.23 × 10^−3^, 3.04 × 10^−3^]
	$u\left( {t = 26236.5{\text{h}} } \right)$	508.19	[328.56, 786.02]
	$m\left( {t = 26236.5{\text{h}} } \right)$	37.29	[29.72, 46.78]
	$u_{c} \left( {t = 26236.5{\text{h}} } \right)$	703.65	[560.81, 882.87]
	$\lambda_{c} \left( {t = 26236.5{\text{h}} } \right)$	1.42 × 10^−3^	[1.13 × 10^−3^, 1.78 × 10^−3^]
	$R\left( {t = 703.65{\text{h}} } \right)$	0.79959735	[0.79959695, 0.79959789]
6#	$\lambda \left( {t = 25503.5{\text{h}} } \right)$	0.92 × 10^−3^	[0.67 × 10^−3^, 1.25 × 10^−3^]
	$u\left( {t = 25503.5{\text{h}} } \right)$	1092.23	[799.32, 1492.48]
	$m\left( {t = 25503.5{\text{h}} } \right)$	25.89	[21.37, 31.38]
	$u_{c} \left( {t = 25503.5{\text{h}} } \right)$	984.96	[812.80, 1193.59]
	$\lambda_{c} \left( {t = 25503.5{\text{h}} } \right)$	1.02 × 10^−3^	[0.84 × 10^−3^, 1.23 × 10^−3^]
	$R\left( {t = 984.96{\text{h}} } \right)$	0.86093534	[0.86093509, 0.86093568]
7#	$\lambda \left( {t = 26266.2{\text{h}} } \right)$	0.74 × 10^−3^	[0.47 × 10^−3^, 1.16 × 10^−3^]
	$u\left( {t = 26266.2{\text{h}} } \right)$	1360.45	[863.00, 2144.63]
	$m\left( {t = 26266.2{\text{h}} } \right)$	19.60	[15.06, 25.49]
	$u_{c} \left( {t = 26266.2{\text{h}} } \right)$	1340.45	[1030.43, 1743.73]
	$\lambda_{c} \left( {t = 26266.2{\text{h}} } \right)$	0.75 × 10^−3^	[0.57 × 10^−3^, 0.97 × 10^−3^]
	$R\left( {t = 1340.45{\text{h}} } \right)$	0.78107615	[0.78107372, 0.78107788]
8#	$\lambda \left( {t = 25429{\text{h}} } \right)$	0.89 × 10^−3^	[0.70 × 10^−3^, 1.14 × 10^−3^]
	$u\left( {t = 25429{\text{h}} } \right)$	1119.26	[874.86, 1431.94]
	$m\left( {t = 25429{\text{h}} } \right)$	29.57	[25.22, 34.67]
	$u_{c} \left( {t = 25429{\text{h}} } \right)$	859.96	[733.49, 1008.25]
	$\lambda_{c} \left( {t = 25429{\text{h}} } \right)$	1.16 × 10^−3^	[0.99 × 10^−3^, 1.36 × 10^−3^]
	$R\left( {t = 859.96{\text{h}} } \right)$	0.78020662	[0.78020588, 0.78020719]
9#	$\lambda \left( {t = 24945.8{\text{h}} } \right)$	0.37 × 10^−3^	[0.29 × 10^−3^, 0.46 × 10^−3^]
	$u\left( {t = 24945.8{\text{h}} } \right)$	2728.02	[2189.23, 3399.41]
	$m\left( {t = 24945.8{\text{h}} } \right)$	17.20	[14.68, 20.15]
	$u_{c} \left( {t = 24945.8{\text{h}} } \right)$	1450.39	[1238.27, 1698.86]
	$\lambda_{c} \left( {t = 24945.8{\text{h}} } \right)$	0.69 × 10^−3^	[0.59 × 10^−3^, 0.81 × 10^−3^]
	$R\left( {t = 1450.39{\text{h}} } \right)$	0.45524699	[0.45523888, 0.45525630]
10#	$\lambda \left( {t = 25836.5{\text{h}} } \right)$	0.42 × 10^−3^	[0.37 × 10^−3^, 0.48 × 10^−3^]
	$u\left( {t = 25836.5{\text{h}} } \right)$	2376.00	[2086.72, 2705.39]
	$m\left( {t = 25836.5{\text{h}} } \right)$	26.87	[24.31, 29.69]
	$u_{c} \left( {t = 25836.5{\text{h}} } \right)$	961.71	[870.19, 1062.86]
	$\lambda_{c} \left( {t = 25836.5{\text{h}} } \right)$	1.04 × 10^−3^	[0.94 × 10^−3^, 1.15 × 10^−3^]
	$R\left( {t = 961.71{\text{h}} } \right)$	0.22301235	[0.22299164, 0.22303484]
11#	$\lambda \left( {t = 24945.3{\text{h}} } \right)$	0.78 × 10^−3^	[0.67 × 10^−3^, 0.91 × 10^−3^]
	$u\left( {t = 24945.3{\text{h}} } \right)$	1278.13	[1093.85, 1493.45]
	$m\left( {t = 24945.3{\text{h}} } \right)$	35.97	[32.19, 40.20]
	$u_{c} \left( {t = 24945.3{\text{h}} } \right)$	693.45	[620.50, 774.99]
	$\lambda_{c} \left( {t = 24945.3{\text{h}} } \right)$	1.44 × 10^−3^	[1.29 × 10^−3^, 1.61 × 10^−3^]
	$R\left( {t = 693.45{\text{h}} } \right)$	0.55097159	[0.55096787, 0.55097459]
12#	$\lambda \left( {t = 24096{\text{h}} } \right)$	1.45 × 10^−3^	[0.94 × 10^−3^, 2.24 × 10^−3^]
	$u\left( {t = 24096{\text{h}} } \right)$	689.82	[446.06, 1066.79]
	$m\left( {t = 24096{\text{h}} } \right)$	29.57	[23.34, 37.46]
	$u_{c} \left( {t = 24096{\text{h}} } \right)$	814.86	[643.22, 1032.30]
	$\lambda_{c} \left( {t = 24096{\text{h}} } \right)$	1.23 × 10^−3^	[0.97 × 10^−3^, 1.55 × 10^−3^]
	$R\left( {t = 814.86{\text{h}} } \right)$	0.65721153	[0.65720935, 0.65721341]

It can be seen from Table 3 that at the last failure the reliability of 6# WT ($R\left( {t = 984.96{\text{h}} } \right) = 0.86093534$) is the largest and the reliability of 10# WT ($R\left( {t = 961.71{\text{h}} } \right) = 0.22301235$) is the smallest. Although the cumulative mean time between failure (CMTBF) of 10# WT about is 961.71 h, it is close to the CMTBF of 6# WT (about 984.96 h). The cumulative failure intensity of 10# WT about is 1.04 × 10^−3^, which is also close to that of 6# WT (about 1.02 × 10^−3^). The instantaneous mean time between failure (IMTBF) of 10# reached about 2376 h, which is about twice that of 6# WT (about 1092.23 h). The instantaneous failure intensity of 6# WT about is 0.92 × 10^−3^, which is about twice that of 10# WT (about 0.42 × 10^−3^). However, because at the time of the initial data collection, the initial failure intensity of 10# WT (about 1.09 × 10^−3^) is the largest, making it the least reliable after the last failure. In contrast, the initial failure intensity of 6# WT (about 0.04 × 10^−3^) is the smallest. Besides, although both the CMTBF (about 1450.39 h) and IMTBF (about 2728.02 h) of 9# WT are the longest, respectively. Its initial failure intensity (0.26 × 10^−3^) is about 4 times that of 6# WT, which makes 9#WTs has less reliable than 6# WT. It shows that the initial failure intensity should be considered in the reliability modelling of WT, which will make the reliability evaluation results closer to the practical engineering. The scheduled time for preventive maintenance of WT can be adjusted based on the mean time between failures.

## Conclusion

In this paper, the 3-BIP was used as the benchmark failure intensity function to modify the LPIM, and the modelling of the reliability of WT with an imperfect repair was conducted. In the modelling, engineering practice conditions such as the failure intensity not being zero at the beginning of observation of WT, the failure intensity increasing with increasing service time, and approaching a constant asymptotically were considered. The results of the case analysis and comparison indicated that the proposed model is a good fit for the WT failure time data. The reliability indices obtained took into account the maintenance history of different WTs and studied the change rule of mean time between failures with service age. The estimated reliability indices can be used to optimize the WT operation and maintenance (O&M) strategies. The preventive maintenance periods of the WTs can are adjusted according to the estimated mean time between failures under a given service time, rather than giving all WTs a unified fixed maintenance period. The proposed method can serve as a guide for modelling and estimating the reliability of other repairable equipment considering imperfect repair effect. But the proposed method did not account for the effect of the difference between failure time data for different failure causes on the estimation of reliability. Also, this issue will be explored in the future.

## Supplementary Information

Supplementary Information.

## Data Availability

All data generated or analyzed during this study are included in this published article.
