# Supplementary material for: Reliability modelling and evaluating of wind turbine considering imperfect repair
Source: Sci Rep. 2023 Apr 1;13:5323. doi: 10.1038/s41598-023-32575-8 (PMC10067845; doi:10.1038/s41598-023-32575-8)
Supplement: Supplementary file 1 — Supplementary Information. [file 41598_2023_32575_MOESM1_ESM.docx]

**Appendix**

$-\frac{\partial^{2}\ln L}{\partial\alpha^{2}}=\frac{n}{\alpha^{2}}$(A1)

$\begin{matrix} -\frac{\partial^{2}\ln L}{\partial\beta^{2}}\text{=}-\sum_{i=1}^{n} \left\{\frac{\exp\left(-\frac{t_{i}+\varepsilon}{\beta}\right)\left(t_{i}+\varepsilon\right)^{2}}{\beta^{4}\left[\exp\left(-\frac{t_{i}+\varepsilon}{\beta}\right)-1\right]}-\frac{2\exp\left(-\frac{t_{i}+\varepsilon}{\beta}\right)\left(t_{i}+\varepsilon\right)}{\beta^{3}\left[\exp\left(-\frac{t_{i}+\varepsilon}{\beta}\right)-1\right]}-\frac{\exp\left[-\frac{2\left(t_{i}+\varepsilon\right)}{\beta}\right]\left(t_{i}+\varepsilon\right)^{2}}{\beta^{4}\left[\exp\left(-\frac{t_{i}+\varepsilon}{\beta}\right)-1\right]^{2}}\right\} \\ +\alpha\sum_{i=1}^{n} \exp\left[\left(i-1\right)\gamma\right]\left[\exp\left(-\frac{t_{i}+\varepsilon}{\beta}\right)\frac{\left(t_{i}+\varepsilon\right)^{2}}{\beta^{3}}-\exp\left(-\frac{t_{i-1}+\varepsilon}{\beta}\right)\frac{\left(t_{i-1}+\varepsilon\right)^{2}}{\beta^{3}}\right] \end{matrix}$(A2)

(A3)

$\begin{matrix} -\frac{\partial^{2}\ln L}{\partial\varepsilon^{2}}=-\sum_{i=1}^{n} \left(\frac{\exp\left(-\frac{t_{i}+\varepsilon}{\beta}\right)}{\beta^{2}\left[\exp\left(-\frac{t_{i}+\varepsilon}{\beta}\right)-1\right]}-\frac{\exp\left[-\frac{2\left(t_{i}+\varepsilon\right)}{\beta}\right]}{\beta^{2}\left[\exp\left(-\frac{t_{i}+\varepsilon}{\beta}\right)-1\right]^{2}}\right) \\ +\alpha\sum_{i=1}^{n} \exp\left[\left(i-1\right)\gamma\right]\left[\frac{1}{\beta}\exp\left(-\frac{t_{i}+\varepsilon}{\beta}\right)-\frac{1}{\beta}\exp\left(-\frac{t_{i-1}+\varepsilon}{\beta}\right)\right] \end{matrix}$(A4)

$-\frac{\partial^{2}\ln L}{\partial\alpha\partial\beta}=\sum_{i=1}^{n} \exp\left[\left(i\text{-}1\right)\gamma\right]\left[\exp\left(-\frac{t_{i}+\varepsilon}{\beta}\right)\text{+}\frac{t_{i}+\varepsilon}{\beta}\exp\left(-\frac{t_{i}+\varepsilon}{\beta}\right)\right.\left.-\exp\left(-\frac{t_{i-1}+\varepsilon}{\beta}\right)-\frac{t_{i-1}+\varepsilon}{\beta}\exp\left(-\frac{t_{i-1}+\varepsilon}{\beta}\right)\right]$(A5)

$-\frac{\partial^{2}\ln L}{\partial\alpha\partial\gamma}=\sum_{i=1}^{n} \left(i-1\right)\exp\left[\left(i-1\right)\gamma\right]\left[t_{i}-t_{i-1}+\beta\exp\left(-\frac{t_{i}+\varepsilon}{\beta}\right)-\beta\exp\left(-\frac{t_{i-1}+\varepsilon}{\beta}\right)\right]$(A6)

$-\frac{\partial^{2}\ln L}{\partial\alpha\partial\varepsilon}=-\sum_{i-1}^{n} \exp\left[\left(i-1\right)\gamma\right]\left[\exp\left(-\frac{t_{i}+\varepsilon}{\beta}\right)-\exp\left(-\frac{t_{i-1}+\varepsilon}{\beta}\right)\right]$(A7)

$-\frac{\partial^{2}\ln L}{\partial\beta\partial\gamma}=\alpha\sum_{i=1}^{n} \left\{\left(i-1\right)\exp\left[\left(i-1\right)\gamma\right]\right.\cdot\left.\left[\exp\left(-\frac{t_{i}+\varepsilon}{\beta}\right)+\frac{t_{i}+\varepsilon}{\beta}\exp\left(-\frac{t_{i}+\varepsilon}{\beta}\right)-\exp\left(-\frac{t_{i-1}+\varepsilon}{\beta}\right)-\frac{t_{i-1}+\varepsilon}{\beta}\exp\left(-\frac{t_{i-1}+\varepsilon}{\beta}\right)\right]\right\}$(A8)

$\begin{matrix} -\frac{\partial^{2}\ln L}{\partial\beta\partial\varepsilon}=-\sum_{i=1}^{n} \left\{\frac{\exp\left(-\frac{t_{i}+\varepsilon}{\beta}\right)}{\beta^{2}\left[\exp\left(-\frac{t_{i}+\varepsilon}{\beta}\right)-1\right]}-\frac{\mathrm{e}\left(t_{i}+\varepsilon\right)\mathrm{xp}\left(-\frac{t_{i}+\varepsilon}{\beta}\right)}{\beta^{3}\left[\exp\left(-\frac{t_{i}+\varepsilon}{\beta}\right)-1\right]}+\frac{\left(t_{i}+\varepsilon\right)\exp\left[-\frac{2\left(t_{i}+\varepsilon\right)}{\beta}\right]}{\beta^{3}\left[\exp\left(-\frac{t_{i}+\varepsilon}{\beta}\right)-1\right]^{2}}\right\} \\ +\alpha\sum_{i=1}^{n} \exp\left[\left(i-1\right)\gamma\right]\left[\frac{\left(t_{i-1}+\varepsilon\right)\exp\left(-\frac{t_{i-1}+\varepsilon}{\beta}\right)}{\beta^{2}}-\frac{\left(t_{i}+\varepsilon\right)\exp\left(-\frac{t_{i}+\varepsilon}{\beta}\right)}{\beta^{2}}\right] \end{matrix}$(A9)

$-\frac{\partial^{2}\ln L}{\partial\gamma\partial\varepsilon}=-\alpha\sum_{i-1}^{n} \left(i-1\right)\exp\left[\left(i-1\right)\gamma\right]\left[\exp\left(-\frac{t_{i}+\varepsilon}{\beta}\right)-\exp\left(-\frac{t_{i-1}+\varepsilon}{\beta}\right)\right]$(A10)

$\frac{\partial\lambda\left(t\right)}{\partial\alpha}=\frac{\exp\left(-\frac{t+\varepsilon}{\beta}\right)-1}{\alpha\gamma t+\alpha\beta\gamma\exp\left(-\frac{t+\varepsilon}{\beta}\right)-\alpha\beta\gamma\exp\left(-\frac{\varepsilon}{\beta}\right)-1}+\frac{\left[\alpha-\alpha\exp\left(-\frac{t+\varepsilon}{\beta}\right)\right]\left[\gamma t+\beta\gamma\exp\left(-\frac{t+\varepsilon}{\beta}\right)-\beta\gamma\exp\left(-\frac{\varepsilon}{\beta}\right)\right]}{\left[\alpha\gamma t+\alpha\beta\gamma\exp\left(-\frac{t+\varepsilon}{\beta}\right)-\alpha\beta\gamma\exp\left(-\frac{\varepsilon}{\beta}\right)-1\right]^{2}}$(A11)

$\begin{matrix} \frac{\partial\lambda\left(t\right)}{\partial\beta}=\frac{\left[\alpha-\alpha\exp\left(-\frac{t+\varepsilon}{\beta}\right)\right]\left[\alpha\gamma\exp\left(-\frac{t+\varepsilon}{\beta}\right)-\alpha\gamma\exp\left(-\frac{\varepsilon}{\beta}\right)+\frac{(t+\varepsilon)\alpha\gamma\exp\left[-\frac{t+\varepsilon}{\beta}\right]}{\beta}-\frac{\alpha\gamma\varepsilon\exp\left(-\frac{\varepsilon}{\beta}\right)}{\beta}\right]}{\left[\alpha\gamma t+\alpha\beta\gamma\exp\left(-\frac{t+\varepsilon}{\beta}\right)-\alpha\beta\gamma\exp\left(-\frac{\varepsilon}{\beta}\right)-1\right]^{2}} \\ +\frac{(t+\varepsilon)\alpha\exp\left(-\frac{t+\varepsilon}{\beta}\right)}{\beta^{2}\left[\alpha\gamma t+\alpha\beta\gamma\exp\left(-\frac{t+\varepsilon}{\beta}\right)-\alpha\beta\gamma\exp\left(-\frac{\varepsilon}{\beta}\right)-1\right]} \end{matrix}$(A12)

$\frac{\partial\lambda\left(t\right)}{\partial\gamma}=\frac{\left[\alpha-\alpha\exp\left(-\frac{t+\varepsilon}{\beta}\right)\right]\left[\alpha t+\alpha\beta\exp\left(-\frac{t+\varepsilon}{\beta}\right)-\alpha\beta\exp\left(-\frac{\varepsilon}{\beta}\right)\right]}{\left[\alpha\gamma t+\alpha\beta\gamma\exp\left(-\frac{t+\varepsilon}{\beta}\right)-\alpha\beta\gamma\exp\left(-\frac{\varepsilon}{\beta}\right)-1\right]^{2}}$(A13)

$\frac{\partial\lambda\left(t\right)}{\partial\varepsilon}=-\frac{\left[\alpha-\alpha\exp\left(-\frac{t+\varepsilon}{\beta}\right)\right]\left[\alpha\gamma\exp\left(-\frac{t+\varepsilon}{\beta}\right)-\alpha\gamma\exp\left(-\frac{\varepsilon}{\beta}\right)\right]}{\left[\alpha\gamma t+\alpha\beta\gamma\exp\left(-\frac{t+\varepsilon}{\beta}\right)-\alpha\beta\gamma\exp\left(-\frac{\varepsilon}{\beta}\right)-1\right]^{2}}-\frac{\alpha\exp\left(-\frac{t+\varepsilon}{\beta}\right)}{\beta\left[\alpha\gamma t+\alpha\beta\gamma\exp\left(-\frac{t+\varepsilon}{\beta}\right)-\alpha\beta\gamma\exp\left(-\frac{\varepsilon}{\beta}\right)-1\right]}$(A14)

$\frac{\partial u\left(t\right)}{\partial\alpha}=-\frac{\gamma t+\beta\gamma\exp\left(-\frac{t+\varepsilon}{\beta}\right)-\beta\gamma\exp\left(-\frac{\varepsilon}{\beta}\right)}{\alpha-\alpha\exp\left(-\frac{t+\varepsilon}{\beta}\right)}-\frac{\left[\exp\left(-\frac{t+\varepsilon}{\beta}\right)-1\right]\left[\alpha\gamma t+\alpha\beta\gamma\exp\left(-\frac{t+\varepsilon}{\beta}\right)-\alpha\beta\gamma\exp\left(-\frac{\varepsilon}{\beta}\right)-\right]}{\left[\alpha-\alpha\exp\left(-\frac{t+\varepsilon}{\beta}\right)\right]^{2}}$(A15)

$\begin{matrix} \frac{\partial u\left(t\right)}{\partial\beta}=-\frac{\alpha\gamma\exp\left(-\frac{t+\varepsilon}{\beta}\right)-\alpha\gamma\exp\left(-\frac{\varepsilon}{\beta}\right)+\frac{(t+\varepsilon)\alpha\gamma\exp\left(-\frac{t+\varepsilon}{\beta}\right)}{\beta}-\frac{\alpha\gamma\varepsilon\exp\left(-\frac{\varepsilon}{\beta}\right)}{\beta}}{\alpha-\alpha\exp\left[-\frac{t+\varepsilon}{\beta}\right]} \\ -\frac{(t+\varepsilon)\alpha\left[\alpha\gamma t+\alpha\beta\gamma\exp\left(-\frac{t+\varepsilon}{\beta}\right)-\alpha\beta\gamma\exp\left(-\frac{\varepsilon}{\beta}\right)-1\right]\exp\left(-\frac{t+\varepsilon}{\beta}\right)}{\beta^{2}\left[\alpha-\alpha\exp\left(-\frac{t+\varepsilon}{\beta}\right)\right]^{2}} \end{matrix}$(A16)

$\frac{\partial u\left(t\right)}{\partial\gamma}=-\frac{\alpha t+\alpha\beta\exp\left(-\frac{t+\varepsilon}{\beta}\right)-\alpha\beta\exp\left(-\frac{\varepsilon}{\beta}\right)}{\alpha-\alpha\exp\left(-\frac{t+\varepsilon}{\beta}\right)}$(A17)

$\frac{\partial u\left(t\right)}{\partial\varepsilon}=\frac{\alpha\gamma\exp\left(-\frac{t+\varepsilon}{\beta}\right)-\alpha\gamma\exp\left(-\frac{\varepsilon}{\beta}\right)}{\alpha-\alpha\exp\left(-\frac{t+\varepsilon}{\beta}\right)}+\frac{\alpha\left[\alpha\gamma t+\alpha\beta\gamma\exp\left(-\frac{t+\varepsilon}{\beta}\right)-\alpha\beta\gamma\exp\left(-\frac{\varepsilon}{\beta}\right)-1\right]\exp\left(-\frac{t+\varepsilon}{\beta}\right)}{\beta\left[\alpha-\alpha\exp\left(-\frac{t+\varepsilon}{\beta}\right)\right]^{2}}$(A18)

$\frac{\partial m\left(t\right)}{\partial\alpha}=-\frac{\gamma t+\beta\gamma\exp\left(-\frac{t+\varepsilon}{\beta}\right)-\beta\gamma\exp\left[-\frac{\varepsilon}{\beta}\right]}{\gamma\left[\alpha\gamma t+\alpha\beta\gamma\exp\left(-\frac{t+\varepsilon}{\beta}\right)-\alpha\beta\gamma\exp\left(-\frac{\varepsilon}{\beta}\right)-1\right]}$(A19)

$\frac{\partial m\left(t\right)}{\partial\beta}=-\frac{\alpha\gamma\exp\left(-\frac{t+\varepsilon}{\beta}\right)-\alpha\gamma\exp\left(-\frac{\varepsilon}{\beta}\right)+\frac{\alpha\gamma(t+\varepsilon)\exp\left(-\frac{t+\varepsilon}{\beta}\right)}{\beta}-\frac{\alpha\gamma\varepsilon\exp\left(-\frac{\varepsilon}{\beta}\right)}{\beta}}{\gamma\left[\alpha\gamma t+\alpha\beta\gamma\exp\left(-\frac{t+\varepsilon}{\beta}\right)-\alpha\beta\gamma\exp\left(-\frac{\varepsilon}{\beta}\right)-1\right]}$(A20)

$\frac{\partial m\left(t\right)}{\partial\gamma}=\frac{\ln\left[\alpha\beta\gamma\exp\left(-\frac{\varepsilon}{\beta}\right)-\alpha\beta\gamma\exp\left(-\frac{t+\varepsilon}{\beta}\right)-\alpha\gamma t+1\right]}{\gamma^{2}}-\frac{\alpha t+\alpha\beta\exp\left(-\frac{t+\varepsilon}{\beta}\right)-\alpha\beta\exp\left(-\frac{\varepsilon}{\beta}\right)}{\gamma\left[\alpha\gamma t+\alpha\beta\gamma\exp\left(-\frac{t+\varepsilon}{\beta}\right)-\alpha\beta\gamma\exp\left(-\frac{\varepsilon}{\beta}\right)-1\right]}$(A21)

$\frac{\partial m\left(t\right)}{\partial\varepsilon}=\frac{\alpha\gamma\exp\left(-\frac{t+\varepsilon}{\beta}\right)-\alpha\gamma\exp\left(-\frac{\varepsilon}{\beta}\right)}{\gamma\left[\alpha\gamma t+\alpha\beta\gamma\exp\left(-\frac{t+\varepsilon}{\beta}\right)-\alpha\beta\gamma\exp\left(-\frac{\varepsilon}{\beta}\right)-1\right]}$(A22)

$\frac{\partial u_{c}\left(t\right)}{\partial\alpha}=\frac{\gamma t\left[\gamma t+\beta\gamma\exp\left(-\frac{t+\varepsilon}{\beta}\right)-\beta\gamma\exp\left(-\frac{\varepsilon}{\beta}\right)\right]}{\ln\left[\alpha\beta\gamma\exp\left(-\frac{\varepsilon}{\beta}\right)-\alpha\beta\gamma\exp\left(-\frac{t+\varepsilon}{\beta}\right)-\alpha\gamma t+1\right]^{2}\left[\alpha\gamma t+\alpha\beta\gamma\exp\left(-\frac{t+\varepsilon}{\beta}\right)-\alpha\beta\gamma\exp\left(-\frac{\varepsilon}{\beta}\right)-1\right]}$(A23)

$\frac{\partial u_{c}\left(t\right)}{\partial\beta}=\frac{\gamma t\left[\alpha\gamma\exp\left(-\frac{t+\varepsilon}{\beta}\right)-\alpha\gamma\exp\left(-\frac{\varepsilon}{\beta}\right)+\frac{\alpha\gamma(t+\varepsilon)\exp\left(-\frac{t+\varepsilon}{\beta}\right)}{\beta}-\frac{\alpha\gamma\varepsilon\exp\left(-\frac{\varepsilon}{\beta}\right)}{\beta}\right]}{\ln\left[\alpha\beta\gamma\exp\left(-\frac{\varepsilon}{\beta}\right)-\alpha\beta\gamma\exp\left(-\frac{t+\varepsilon}{\beta}\right)-\alpha\gamma t+1\right]^{2}\left[\alpha\gamma t+\alpha\beta\gamma\exp\left(-\frac{t+\varepsilon}{\beta}\right)-\alpha\beta\gamma\exp\left(-\frac{\varepsilon}{\beta}\right)-1\right]}$(A24)

$\begin{matrix} \frac{\partial u_{c}\left(t\right)}{\partial\gamma}=\frac{\gamma t\left[\alpha t+\alpha\beta\exp\left(-\frac{t+\varepsilon}{\beta}\right)-\alpha\beta\exp\left(-\frac{\varepsilon}{\beta}\right)\right]}{\ln\left[\alpha\beta\gamma\exp\left(-\frac{\varepsilon}{\beta}\right)-\alpha\beta\gamma\exp\left(-\frac{t+\varepsilon}{\beta}\right)-\alpha\gamma t+1\right]^{2}\left[\alpha\gamma t+\alpha\beta\gamma\exp\left(-\frac{t+\varepsilon}{\beta}\right)-\alpha\beta\gamma\exp\left(-\frac{\varepsilon}{\beta}\right)-1\right]} \\ -\frac{t}{\ln\left[\alpha\beta\gamma\exp\left(-\frac{\varepsilon}{\beta}\right)-\alpha\beta\gamma\exp\left(-\frac{t+\varepsilon}{\beta}\right)-\alpha\gamma t+1\right]} \end{matrix}$(A25)

$\frac{\partial u_{c}\left(t\right)}{\partial\varepsilon}=-\frac{\gamma t\left[\alpha\gamma\exp\left(-\frac{t+\varepsilon}{\beta}\right)-\alpha\gamma\exp\left(-\frac{\varepsilon}{\beta}\right)\right]}{\ln\left[\alpha\beta\gamma\exp\left(-\frac{\varepsilon}{\beta}\right)-\alpha\beta\gamma\exp\left(-\frac{t+\varepsilon}{\beta}\right)-\alpha\gamma t+1\right]^{2}\left[\alpha\gamma t+\alpha\beta\gamma\exp\left(-\frac{t+\varepsilon}{\beta}\right)-\alpha\beta\gamma\exp\left(-\frac{\varepsilon}{\beta}\right)-1\right]}$(A26)

$\frac{\partial\lambda_{c}\left(t\right)}{\partial\alpha}=-\frac{\gamma t+\beta\gamma\exp\left(-\frac{t+\varepsilon}{\beta}\right)-\beta\gamma\exp\left(-\frac{\varepsilon}{\beta}\right)}{\gamma t\left[\alpha\gamma t+\alpha\beta\gamma\exp\left(-\frac{t+\varepsilon}{\beta}\right)-\alpha\beta\gamma\exp\left(-\frac{\varepsilon}{\beta}\right)-1\right]}$(A27)

$\frac{\partial\lambda_{c}\left(t\right)}{\partial\beta}=-\frac{\alpha\gamma\exp\left(-\frac{t+\varepsilon}{\beta}\right)-\alpha\gamma\exp\left(-\frac{\varepsilon}{\beta}\right)+\frac{\alpha\gamma(t+\varepsilon)\exp\left(-\frac{t+\varepsilon}{\beta}\right)}{\beta}-\frac{\alpha\gamma\varepsilon\exp\left(-\frac{\varepsilon}{\beta}\right)}{\beta}}{\gamma t\left[\alpha\gamma t+\alpha\beta\gamma\exp\left(-\frac{t+\varepsilon}{\beta}\right)-\alpha\beta\gamma\exp\left(-\frac{\varepsilon}{\beta}\right)-1\right]}$(A28)

$\frac{\partial\lambda_{c}\left(t\right)}{\partial\gamma}=\frac{\ln\left[\alpha\beta\gamma\exp\left(-\frac{\varepsilon}{\beta}\right)-\alpha\beta\gamma\exp\left(-\frac{t+\varepsilon}{\beta}\right)-\alpha\gamma t+1\right]}{t\gamma^{2}}-\frac{\alpha t+\alpha\beta\exp\left(-\frac{t+\varepsilon}{\beta}\right)-\alpha\beta\exp\left(-\frac{\varepsilon}{\beta}\right)}{\gamma t\left[\alpha\gamma t+\alpha\beta\gamma\exp\left(-\frac{t+\varepsilon}{\beta}\right)-\alpha\beta\gamma\exp\left(-\frac{\varepsilon}{\beta}\right)-1\right]}$(A29)

$\frac{\partial\lambda_{c}\left(t\right)}{\partial\varepsilon}=\frac{\alpha\gamma\exp\left(-\frac{t+\varepsilon}{\beta}\right)-\alpha\gamma\exp\left(-\frac{\varepsilon}{\beta}\right)}{\gamma t\left[\alpha\gamma t+\alpha\beta\gamma\exp\left(-\frac{t+\varepsilon}{\beta}\right)-\alpha\beta\gamma\exp\left(-\frac{\varepsilon}{\beta}\right)-1\right]}$(A30)

$\frac{\partial R\left(\text{t}\right)}{\partial\alpha}=-\frac{\left[t\gamma+\beta\gamma\exp\left(-\frac{t+\varepsilon}{\beta}\right)-\beta\gamma\exp\left(-\frac{\varepsilon}{\beta}\right)\right]\left[\alpha\beta\gamma\exp\left(-\frac{\varepsilon}{\beta}\right)-\alpha\beta\gamma\exp\left(-\frac{t+\varepsilon}{\beta}\right)-\alpha\gamma t+1\right]^{\frac{1}{\gamma}-1}}{\gamma}$(A31)

(A32)

$\begin{matrix} \frac{\partial R\left(\text{t}\right)}{\partial\gamma}=-\frac{\ln\left[\alpha\beta\gamma\exp\left(-\frac{\varepsilon}{\beta}\right)-\alpha\beta\gamma\exp\left(-\frac{t+\varepsilon}{\beta}\right)-\alpha\gamma t+1\right]\left[\alpha\beta\gamma\exp\left(-\frac{\varepsilon}{\beta}\right)-\alpha\beta\gamma\exp\left(-\frac{t+\varepsilon}{\beta}\right)-\alpha\gamma t+1\right]^{\frac{1}{\gamma}}}{\gamma^{2}} \\ -\frac{\left[\alpha t+\alpha\beta\exp\left(-\frac{t+\varepsilon}{\beta}\right)-\alpha\beta\exp\left(-\frac{\varepsilon}{\beta}\right)\right]\left[\alpha\beta\gamma\exp\left(-\frac{\varepsilon}{\beta}\right)-\alpha\beta\gamma\exp\left(-\frac{t+\varepsilon}{\beta}\right)-\alpha\gamma t+1\right]^{\frac{1}{\gamma}-1}}{\gamma} \end{matrix}$(A33)

$\frac{\partial R\left(\text{t}\right)}{\partial\varepsilon}=\frac{\left[\alpha\gamma\exp\left(-\frac{t+\varepsilon}{\beta}\right)-\alpha\gamma\exp\left(-\frac{\varepsilon}{\beta}\right)\right]\left[\alpha\beta\gamma\exp\left(-\frac{\varepsilon}{\beta}\right)-\alpha\beta\gamma\exp\left(-\frac{t+\varepsilon}{\beta}\right)-\alpha\gamma t+1\right]^{\frac{1}{\gamma}-1}}{\gamma}$(A34)

Table A1 Failure time data of WTs

| WTs | failure time/h | | | | | | | | |  |
| --- | --- | --- | --- | --- | --- | --- | --- | --- | --- | --- |
| 1# | 1406.8 | 3038.5 | 4390.2 | 4632 | 6060.7 | 7036 | 7395.2 | 8133 | 8388.5 | 8970 |
|  | 9088 | 9187.8 | 9328 | 9852 | 10336.8 | 10980.8 | 11577.7 | 12164.2 | 12427.6 | 12515 |
|  | 13598 | 15288 | 16377.3 | 16719.5 | 17269.8 | 18308 | 18474.9 | 18957 | 18960 | 18979 |
|  | 20318 | 20840.4 | 22814 | 23776.5 | 25744.8 |  |  |  |  |  |
| 2# | 5146 | 5411.3 | 5722.8 | 6733.7 | 8558 | 8789.8 | 9207.2 | 9336 | 9399.2 | 9526.2 |
|  | 9794.2 | 10157.5 | 10265 | 10449.5 | 10600.5 | 10805.5 | 11059.5 | 11222 | 11934.2 | 13697 |
|  | 14417.3 | 15327.2 | 17631.3 | 17681.8 | 18792 | 18902.5 | 19279.8 | 20438 | 20493.5 | 20777.2 |
|  | 21576.8 | 24247.8 | 24764 | 24795 | 24947 | 25704 |  |  |  |  |
| 3# | 340 | 970 | 1030.5 | 6315.1 | 6913.6 | 6951.9 | 7991.5 | 8016 | 8774.5 | 10330.8 |
|  | 11749.6 | 11945 | 12448.5 | 12788 | 12935.5 | 13636.8 | 13890 | 16194.9 | 16407 | 22387 |
|  | 24398 | 24945.5 |  |  |  |  |  |  |  |  |
| 4# | 1160.8 | 1930 | 4073.6 | 4746 | 5803 | 6976.3 | 7657 | 8057.5 | 10506 | 11132.6 |
|  | 13203.7 | 16159.8 | 16427.7 | 17390.5 | 17669.2 | 18375.5 | 18976.8 | 19261.5 | 20806.5 | 21098 |
|  | 21122.5 | 21143.5 | 21533.8 | 26037.5 |  |  |  |  |  |  |
| 5# | 1339  12172.5 | 3027.5  12490 | 3211.3  12512 | 4920.2  13022.7 | 5672.4  14355.5 | 6513.2  16507.3 | 6586  16560 | 6861  17385.5 | 10977.6  17781 | 12122.7  18219 |
|  | 18264 | 18567.2 | 19416 | 19547 | 19698.5 | 19704 | 19937.8 | 20625.5 | 21161 | 21211.3 |
|  | 21275.4 | 21324.2 | 23272 | 25663.5 | 25752 | 26236.5 |  |  |  |  |
| 6# | 149.5 | 2688.2 | 6444 | 6602 | 6947 | 7001.7 | 8222.5 | 10272 | 11691.8 | 11760 |
|  | 12074.5 | 12809.3 | 12987.7 | 14364.2 | 14437.5 | 16080 | 16380 | 17632 | 18070.5 | 19823.5 |
|  | 20677.2 | 22120.7 | 22357.2 | 22368 | 25503.5 |  |  |  |  |  |
| 7# | 347.8 | 5149.5 | 7905.3 | 8016 | 8767.2 | 10678 | 11419.2 | 12718.5 | 12777.5 | 12861.6 |
|  | 14038.4 | 18066.8 | 18377.9 | 19154 | 21089 | 22922.2 | 24201.7 | 24288.2 | 25540 | 25718 |
|  | 26266.2 |  |  |  |  |  |  |  |  |  |
| 8# | 414 | 2050.2 | 6117.7 | 6315.1 | 6699.2 | 7558 | 7865 | 8068 | 8460.5 | 9187.5 |
|  | 9343.5 | 10264 | 10432 | 10728 | 11383 | 12353 | 12593.5 | 13259 | 14407.5 | 17664 |
|  | 18902.5 | 19787.8 | 19989.5 | 20502.6 | 20905 | 21065 | 21206.7 | 21777 | 25429 |  |
| 9# | 184 | 339 | 3389.8 | 3527.7 | 3696 | 7911.3 | 8352 | 8944 | 9151.3 | 10714 |
|  | 12270.5 | 17597.8 | 17795 | 20269.8 | 20346.8 | 21188.5 | 22821 | 24112 | 24945.8 |  |
| 10# | 368 | 816 | 1160 | 1341.8 | 2942.4 | 3303.2 | 3474.5 | 3518 | 3696 | 4699.7 |
|  | 5294.3 | 5462.4 | 6874.5 | 7267.5 | 12010.8 | 12681.2 | 13357.9 | 13392 | 13570.5 | 16380.8 |
|  | 19002.6 | 19258.5 | 19570.2 | 19743 | 20415 | 21285.8 | 22878.5 | 23082.2 | 24232 | 25836.5 |
| 11# | 255.3 | 806.8 | 1008.1 | 1188.1 | 1340.4 | 3601.6 | 3990 | 4040 | 4663.8 | 4761.8 |
|  | 4800 | 4923 | 8082 | 8659.2 | 8826.2 | 9185.8 | 9370 | 9432.5 | 9940 | 10272 |
|  | 11248.5 | 12746.2 | 13341 | 13648.2 | 16720 | 16962.2 | 17224.5 | 17280 | 18305.3 | 18457.5 |
|  | 18690.5 | 18882.5 | 18977.3 | 19023.2 | 22205 | 24026 | 24945.3 |  |  |  |
| 12# | 3376.5 | 3755 | 5294.3 | 5856 | 8060 | 8633.5 | 8757.3 | 8760 | 8856 | 10257.6 |
|  | 10528.1 | 11406.5 | 11732 | 13096 | 14011.3 | 14081 | 14291.5 | 17293 | 17658.4 | 17997 |
|  | 18977.5 | 18994 | 19175.5 | 19237.7 | 20973.8 | 21616.8 | 21804.5 | 23088 | 24096 |  |

Fig.A1. TTT graph of WTs
